# Case of Rabies Canina, or Hydrophobia, with Remarks

**Published:** 1805-02-01

**Authors:** Samuel Argent Bardsley

**Affiliations:** Physician to the Manchester Infirmary, Lunatic Hospital, &c.


### Case of Rabies Canina, or Hydrophobia, with Remarks. *Communicated by* Samuel Argent Bardsley, M.D. *Physician to the Manchester Infirmary, Lunatic Hospital, &c.*

On the 31st of August, 1803, at eleven o'clock, A. M. I was desired to visit John Warren, ætat. 16, who had just been admitted, as a case of emergency, into the Manchester Infirmary. I learned the following particulars from the patient, and his parents who accompanied him. The latter were examined apart, as I wished to avoid exciting any suspicion in the patient of the nature of his malady, which had already been represented to me as a supposed case of Hydrophobia.

To the best of the united recollection of the parties, the patient was bitten by a strange dog, about four months ago, (sometime in May) on the outside of the right hand, near to the first joint of the little finger, while playing with his companions in the dusk of the evening. The animal suddenly darted on one side, and seized him by the hand, which it held for some time in its mouth. A severe wound wound was the consequence of the bite, which continued open for about ten days, and then healed with little difficulty. No suspicion occurring to the boy or his friends, of the animal being mad, the wound was dressed with common cerate, and no internal medicine exhibited.

On Saturday, August 27, the patient first complained of coldness, and a slight numbness, extending from the cicatrix of the wounded hand along the arm, up to the shoulder and neck of the same side. General rigors succeeded to this local sense of coldness, attended with loss of appetite and depression of spirits. The latter symptom was more particularly noticed, as the boy had enjoyed general good health, and was of an active, lively disposition. He passed a restless night, and the next morning (the 28th) experienced, for the first time, when attempting to drink his tea at breakfast, a pain and stricture about the throat, and great difficulty in swallowing the liquid. This symptom was followed by restlessness, great anxiety, and an aversion to keep the body in a recumbent posture. Considerable thirst and clamminess of the mouth were felt; to remove which, the patient made several fruitless efforts to get down water, and other liquids. He was distressed by the air blowing upon him, and complained of its chilling effects, although the weather was close and warm. He had passed the whole of the interval from his seizure on the 27th, till his admission on the 31st, without sleep, and in either a standing or sitting posture. Some little solid food had been swallowed, but with scarcely any relish or appetite.

From this recital, there could scarcely be entertained any doubt of the nature of the patient's disorder. Having attended upon two fatal cases of genuine hydrophobia, and consequently witnessed the peculiarity of voice, gesture, and manner that characterise (independently of the dread of liquids) this disease in all its horrid calamity, (and which must for ever baffle the power of language adequately to describe) it only required a few moments attentive observation to decide upon the character of his complaint. At the time of his admission, (eleven o'clock, A. M.) his countenance expressed great distress and anxiety. The eyes wandered, at times, with apprehension and distrust, from one object to another; and occasional twitchings might be perceived in the muscles of the face and throat. He was restless and impatient, and, indeed, no persuasions could prevail upon him to sit steadily, for a minute, upon one seat, seat. On attempting to drink some wine and water, he was seized, on the liquid touching his lips, with a sense of suffocation and violent spasmodic contraction of the muscles about the throat and fauces; the larynx became suddenly inflated and as suddenly depressed, and the muscles of the neck seemed to swell and enlarge in a surprising manner. By resolute perseverance, however, a small portion of the liquid was forced down. He ate a little solid food with tolerable facility. But upon barely mentioning, that a vessel of water was to be filled in the adjoining apartment, he experienced a renewal of the oppression at the præcordia, and gasped for breath with vehement struggles. His pulse 120, somewhat feeble, and irregular. His skin moderately warm and rather dry; but every now and then, he described himself to be afflicted with a glow of heat, "as if on fire," (to use his own expression) which began in the legs, and gradually extending upwards, until the whole body was in a flame. This sensation of extreme and peculiar heat was followed by slight rigors; yet, what is worthy of remark, the temperature of the skin was not augmented or diminished, during either the hot or cold stage. Being desirous to ascertain whether my patient suspected the nature of his disorder, I put some questions to him, concerning the origin of the accident. But I found, that although the circumstance of his having been injured by the dog had sunk deep into his mind, yet it was unaccompanied with any notion of a connection between the accident and his present malady. The degree of terror, arising from the suddenness of the attack, during the obscuring of the approach of night, had affected his imagination so strongly, that he emphatically, and with a look of disgust and horror, told me, that the animal "rushed upon him suddenly like a black spirit, and made him shriek with fright;" but that his alarm had abated when he could discern that it was only a black dog, which he had before seen in the neighbourhood. He now complained of an uneasy sensation in the wounded hand, which, darting along the arm, fixed itself in the throat and breast. No discolouration, or tumefaction, could be discerned about the cicatrized wound, where these pains originated. A copious flow of saliva occasioned him to spit out frequently; and although the tongue appeared clean, he complained of a fetid taste in his mouth, and bad smell in his nostrils.

From the history of the case, previous to the patient's admission into the Infirmary and the present state of the symptoms, symptoms, little or no chance of success could be expected from any medical treatment; for the disorder might be fairly considered as advanced to its last stage. But notwithstanding that the patient's doom might be pronounced as nearly certain; yet it was felt an urgent duty to attempt every thing with promptitude and diligence, that might afford the least chance of relief. One grain of opium was given immediately on his entrance into the ward, and three pills composed of bread-crumb moistened with the spt. ammon. pur.* were ordered to be swallowed every half hour. A caustic was applied to the cicatrix of the wound; and as the fame of Galvanism, in the cure of hydrophobia, had gone abroad, it was thought proper not to neglect giving this remedy a trial. For this purpose I solicited the aid of my friend, Mr. William Henry, (whose celebrity as a chemist and philosopher is well known) in administering from a voltaic pile in his possession, Galvanic shocks through various parts of the body; but chiefly the head, throat, and breast. The apparatus consisted of sixty plates; but no very perceptible effects followed its application, except when the conductors were applied to the temples. Then, and then only, did the patient discover any strong signs of his being affected by the Galvanic influence. During the operation, which lasted a considerable time, the pulse was somewhat accelerated, but no other change could be perceived. The patient was now conducted to a slipper-bath, heated to ninety-six degrees. He was greatly appalled at the sight of the water, and could not be persuaded, for a length of time, to lie down in the bath, or suffer the fluid to rise higher than his chest. His fears of suffocation, when the water approached the præcordia, amounted to an agony; and being threatened with violent convulsions, he was speedily removed from his distressing situation. He now (two o'clock, P. M.) swallowed a third pill, containing thirty

* I was induceed to make trial of this remedy, from its efficacy in subduing the deleterious effects produced by the bite of the cobra de capello. The close analogy to be observed between the symptoms produced by the bite of a rabid dog, and those whieh followed a similar injury inflicted by the cobra de capello, might warrant the supposition of an identity in the nature of their respective causes; and consequently the plan of cure, which had succeeded in the one case, might rationally be expected to answer in the other. This is further illustrated in my paper "On Canine and spontaneous Hydrophobia, published in the 4th volume of the Manchester Society's Memoirs.

ty drops of the pure caustic alkali, with little or no difficulty. Half an ounce of tinct. opii mixed with the yolk of egg, was ordered to be rubbed into the inside of the legs and thighs, and his pills to be repeated as before directed. A consultation had been called in the forenoon, of Dr. Percival, physician extraordinary, and all the rest of the faculty belonging to the institution; but could not be held till five o'clock, P. M.; at which time the whole body met; and after due consideration of all the circumstances of the case, the following plan was ordered to be pursued. One grain of cantharides* finely powdered, and made up into a pill, with mucilage of gum arabic and crumb of bread, was to be immediately given; and repeated every two hours, until a strangury, or some other sign of irritation was excited. The patient was also ordered to be placed on an insulated bed and exposed to the electrical influence, by the medium of a chain connected with the conductor of the machine and the body of the patient, for the space of one hour. Sparks were likewise to be drawn from the throat and parts adjacent for twenty minutes, and both these processes to be twice repeated before twelve o'clock. As the administration of opium, by friction, had produced a drowsiness, and a suspension of his sufferings for a few minutes, it was to be renewed twice during the time of the patient's exposure to the effects of electricity. In half an hour from the period of the electrical machine being put in motion, he became more calm and tractable, and expressed a desire to drink some water, which he was able to perform with comparative ease and readiness; and with no small marks of pleasure, and even triumph. After the frictions had been completed, he could lie down for ten minutes together, and did not start up in his usual hurried and distressed manner. On visiting him again, at twelve o'clock the same evening, I found he

---

* I ventured to propose the internal exhibition of cantharides, with a view to bring on a strangury, from an acknowledged principle in the animal economy, viz. that one irritation already existing may be cured by exciting a counter-irritation. The use of cantharides in hydrophobia is mentioned by Morgagni, and has been lately in vogue among several German practitioners for a similar purpose. The safety and utility of the practice, in treating some desperate cases of tetanus, led Dr. Mease to recommend its trial in hydrophobia, which he (justly I think) deemed to be strictly allied to tetanus, as a disease of nervous irritation and debility. Any kind of practice, however, which is founded on rational analogy, may, with propriety, be attempted in the cure of, what may be hitherto termed, an irremediable disease.

he had passed the interval from my last visit, with alternate exacerbations and remissions of the more violent symptoms. He had taken four doses of the pills with cantharides, and had felt much relieved by an emollient glyster, that had been provisionally directed. This injection had procured a plentiful discharge from the bowels of regular fæces. For the last hour, he had been subjected (in the usual manner) to electricity, by the aid of a very powerful machine, which was directed by a professed and skilful electrician; and strong sparks were also drawn from the throat and præcordia; but no shocks were attempted to be given. The sparking now seemed to increase his agitation, and was therefore soon discontinued. He complained of a slight pain in the pubis; but without any strangury. As this effect had not been produced, and the stomach and bowels did not appear to suffer from the use of the cantharides, a repetition of the pill every hour was directed, until some manifest impression had taken place. The opiate friction to be continued; and as the patient complained of extreme thirst and dryness of the fauces, a mixture of nitric acid, sugar and water, was prescribed as a common drink. Although the patient could both swallow some small portion of liquids, and bear a recumbent posture for many minutes, yet his strength was fast declining. The pulse had become feeble and irregular, and all his motions and gestures denoted increased debility. He gasped, at intervals, with eagerness for breath, and a remarkable lateral motion of the jaw might be occasionally perceived, which seemed partly to arise from all the muscles subservient to deglutition being forcibly exerted in procuring a flow of saliva, to relieve the parched state of the mouth and fauces; and partly from an effort to assist his impeded respiration, which, at times, was increased almost to suffocation. This symptom of stricture at the breast engaged his constant attention; and he seemed to wish for a wider expanse of room and air to breathe in. He expressed the utmost horror at being requested to blow his nostrils; nor would he suffer his face to be touched by his own, or any other person's hands. Indeed, he often cried out, "I shall be choaked," if an attempt was made to wipe away the mucus from his mouth and nostrils. After remaining with him about an hour, I found the spasmodic constriction at the throat continued to increase, and that slight convulsions assailed the muscles of the neck, breast and arms. He still felt shooting pains dart rapidly along the injured hand; hand and arm to the throat and side; but still no visible alteration in the wounded part could be discovered. His timidity had increased; and he examined every face and object with a suspicious eye. The pulse was tremulous and irregular, and exceeded 140 strokes in the minute. His voice hoarse, indistinct, and wailing; could scarcely articulate the expressions of despair. He soon sunk into a drowsy torpid state; but, at intervals, uttered delirious shrieks, attended with convulsive spasms, threatening instant destruction. The termination of his sufferings was now evidently approaching. His struggles for breath became fainter and fainter. The extremities felt cold, his eyes were dim and sunk, and a cold sweat bedewed his face and neck. No hope remaining of any medical relief, he was committed solely to the pious and affectionate assiduities of his sorrowful parents, (neither of whom had scarcely ever quitted his bedside since his entrance into the infirmary) and in their presence he expired, at half past four o'clock in the morning, (September 1, being the sixth day from his first seizure, and the fifth from the commencement of the dread of liquids,) without suffering any violently convulsive or painful struggles.

### *Appearances on Dissection.*

The anxious wish of the parents, that the body might be disfigured as little as possible, precluded any farther than a partial inspection. They were particularly averse to the head being opened; as, in that case, they imagined their prejudiced neighbours would discover what had been done, and blame them for permitting the same.

The dissection took place in the presence of several gentlemen connected with the infirmary, in the forenoon of the same day on which the patient died. The body appeared black in several places, and the eyes remarkably small and dim, and buried deeply within their sockets.

The principal appearances were as follow:

The muscles of the œsophagus and trachea exhibited no appearances of inflammation.

Trachea and lungs:—The inner surface of trachea, just before its bifurcation and entrance into the lungs, in the opinion of one or two of the gentlemen present, was slightly inflamed. But the indications of inflammation were certainly of an equivocal and dubious kind. Something like dots, or strokes of red blood, were discernible upon a small portion of the inner membrane; but they were neither numerous nor distinct. No adhesions to the pleura, nor turgescency of the vessels of the lungs, could be perceived. The heart and pericardium were free from disease.

Stomach.—As in the other two instances which I had inspected, inflammation, in a greater or lesser degree, was discovered upon the inner coats of this organ; and the same phænomenon having been observed in perhaps the majority of other cases, great attention was paid to the condition of this viscus. No external appearance of disease could be traced; nor, when its contents had been discharged, (which amounted to about three ounces of a dark coloured and somewhat tenacious fluid) and its coats gently cleansed with warm water, and exposed to view by a longitudinal division, from the cardia to the pylorus: was there any trace of inflammation to be discovered—small particles of the cantharides lay scattered within its rugæ or folds; but even this irritating substance had not induced an inflammatory appearance.

The viscera of the abdomen were perfectly sound, and exhibited no uncommon appearance.

### Remarks.

I shall venture to make a few remarks on this case, with a reference to the character and treatment of hydrophobia. The history of the symptoms, and its fatal termination, leave no room to doubt of its being an instance of rabies canina, or rabid hydrophobia. No intelligence, indeed, could be gained concerning the real state of the dog; but the symptoms of pain, numbness, and coldness, that originated in the cicatrix of the patient's wounded hand, four months after the bite; together with the speedy accession of hydrophobia, and the train of other characteristic symptoms which succeeded, seem to me quite decisive on the point in question. Among the symptoms enumerated in the history of this case, one may be noticed which did not appear in those instances that have fallen immediately under my observation; nor has it indeed been distinctly remarked by writers on the disease. I allude to the very troublesome sensation of heat, or burning, which the patient described, as extending from his legs throughout the whole frame; and yet unaccompanied by any sensible demonstration of increased temperature. Does not this fact strengthen the supposition, that the canine virus operates upon the nervous system? For in many hysterical and nervous cases, such sensations are often complained of; yet, neither in these, nor in the instance under consideration, tion, did it appear, that the action of the vascular system was in any way affected during this extraordinary heat or glow: moreover, the sensation is transitory and fluctuating in both instances.

I had occasion to remark, in former cases, some degree of that lateral and, at times, depressed motion of the jaw, which formed a striking feature in this patient's complaint. That it arises from the causes before enumerated, I think, is highly probable. But what is well worthy of observation, not only in this, but in every other case of genuine hydrophobia, (that I have either seen or heard of) the existence of stricture about the throat and præcordia, invariably constitutes one of the primary symptoms of this baleful malady; increases with its increase, and attends the patient till nearly the last moment of his existence. Happy would it be for humanity, and highly honourable to the medical art, if a remedy could be discovered for this justly reputed *opprobrium* of medicine! It is, however, somewhat consolatory to find, that opiate frictions, and the use of electricity (in the manner already pointed out) seemed to possess the power of abating the irritability of the system, and producing a transient mitigation of the patient's sufferings. Considering the very late period of the disease at which these remedies were tried, no very sanguine hopes could be entertained of their being useful, even as *palliative* means; but may not more permanently beneficial effects be derived from their exhibition in a very early state of the disorder? The administration of two such powerful medicines, as the pure caustic alkali and cantharides in substance, seems at least to be safe, if not useful, from what has been already stated. Most unquestionably, if it should ever fall to my lot to treat a third case of so distressing and fatal a malady, I shall be tempted to try safe, yet active doses of the arsenical solution (as the most powerful tonic in the whole range of the materia medica) along with wine, opiate frictions, and electricity. I am the more confirmed in the hope of utility from this practice, as it met with the approbation and recommendation of my highly respected and much lamented friend, the late Dr. Percival. Sanctioned by his authority, medical practitioners may probably be induced to make trial of this method of cure:—a method that, in part, has certainly succeeded in alleviating some of the symptoms of hydrophobia; but which, indeed, is chiefly recommended by its supposed agreement with the rational principles of analogical induction.

*Manchester, Dec.* 11, 1804.

